# A Joint Method Based on Time-Frequency Distribution to Detect Time-Varying Interferences for GNSS Receivers with a Single Antenna

**DOI:** 10.3390/s19081946

**Published:** 2019-04-25

**Authors:** Qingshui Lv, Honglei Qin

**Affiliations:** School of Electronic and Information Engineering, Beihang University, Beijing 100191, China; lvqingshui0501@163.com

**Keywords:** GNSS, time-varying interference, single antenna, RSPWVD, Hough transform

## Abstract

In this paper, a joint method combining Hough transform and reassigned smoothed pseudo Wigner-Ville distribution (RSPWVD) is presented to detect time-varying interferences with crossed frequency for a Global Navigation Satellite System (GNSS) receiver with a single antenna. The proposed method can prevent the cross-term interference and detect the time-varying interferences with crossed frequency which cannot be achieved by the classical time-frequency (TF) analysis with the peak detection method. The actual performance of the developed method has been evaluated by experiments with conditions where the real BeiDou system (BDS) B1I signals are corrupted by the simulated chirp interferences. The results of experiments show that the introduced method is effectively able to detect chirp interferences with crossed frequency and provide the same root mean square errors (RMSE) of the parameter estimation for chirp one and the improved initial frequency estimation for chirp two compared with the Hough transform of Wigner-Ville distribution (WVD) when the jamming to noise ratio (JNR) equals or surpasses 4 dB.

## 1. Introduction

At present, GNSS receivers with a single antenna are threatened by a serious jamming environment where many GNSS receiver failures occurred [1,2,3], limiting the GNSS applications. As a result, the techniques used to detect and mitigate interference effects have become an increasingly important issue and can be divided into the automatic gain control (AGC) method [4], digital signal processing methods [5,6,7,8,9] and receiver methods [10,11] from GNSS signal processing chains.

The AGC acting as an adaptive variable gain amplifier adjusts the input signal level to the analog-to-digital converter (ADC) input range [10,12]. Therefore, the AGC gain variation can be utilized to detect interference, especially continuous wave interference (CWI) and pulsed interference (PI). However, there are not enough effective quantized bits for ADC to realize the gain variation range of AGC for a common GNSS receiver.

From the GNSS receiver, the position accuracy and effective carrier to noise density ratio are commonly adopted to assess the impact of interference on GNSS receivers [13,14]. However, both of them rely on a particular GNSS receiver performance. If the jamming power surpasses the spread spectrum gain of GNSS causing the GNSS receiver not to work, the corresponding algorithm will fail.

For the digital signal processing methods, they include spatial domain methods [5], spatial-temporal domain methods [15,16,17], time domain methods [6], frequency domain methods [7], and time-frequency (TF) domain methods [8,9]. Time domain techniques as well as frequency domain techniques cannot completely describe the nature of time-varying signals.

Spatial domain techniques [5] and spatial-temporal domain methods assume that the GNSS signal angle of arrival (AOA) and the antenna model are known for the receiver. The antenna model is sensitive to AOA estimation error and can cause a high computational complexity. In addition, an antenna array is required, which is difficult to install on small devices.

TF domain methods adopt the classical TF analysis including short-time Fourier transform (STFT), Wigner-Ville distribution (WVD) and RSPWVD [8], which illustrate that jamming signals usually focus their energy in a finite area of the TF plane, while noise power extends over the whole TF plane [9]. However, these TF methods cannot deal with the time-varying interferences with crossed frequency by the peak detection method [18]. In this paper, a joint method combining Hough transform and RSPWVD is depicted to detect chirp interferences with crossed frequency for GNSS receivers with a single antenna. The analytic expression of initial frequency estimation and chirp rate estimation is presented and the double threshold detection is proposed as well. In addition, the effect of sweep period on the estimation of initial frequency and chirp rate has been analyzed.

## 2. Signal and System Model

The signals received from an antenna of a GNSS receiver pass through the radio frequency (RF) front-end and are down-converted to intermediate frequency (IF). Before the ADC, they can be written as

(1) $$X_{IF}\left(t\right)=\sum_{l=1}^{N_{s}}S_{IF,l}\left(t\right)+J_{IF}\left(t\right)+N\left(t\right)$$

where N(t) is the additive white Gaussian noise term with two-sided power spectral density $N_{0}/2$ and zero mean, $J_{IF}\left(t\right)$ is the jamming signal. $N_{s}$ is the number of visible satellites, $S_{IF,l}\left(t\right)$ is the signal transmitted by the *l*th satellite, which can be defined

(2) $$S_{IF,l}\left(t\right)=\sqrt{2A_{l}}C_{l}\left(t-\tau_{l}\right)D_{l}\left(t-\tau_{l}\right)\cos\left[2\pi \left(f_{IF}+f_{d,l}\right)t+\varphi_{IF,l}\right]$$

where,

- $A_{l}$ is the received GNSS signal power from the *l*th satellite;
- $C_{l}\left(t-\tau_{l}\right)$ is the pseudorandom noise sequence, and $\tau_{l}$ is the code phase delay;
- $D_{l}\left(t-\tau_{l}\right)$ is the navigation data message signal;
- $f_{IF}$ is GNSS signal intermediate frequency;
- $f_{d,l}$ is the Doppler-affected frequency;
- $\varphi_{IF,l}$ is the carrier phase delay.

The jamming signal $J_{IF}\left(t\right)$ defined in Equation (1) is redefined

(3) $$J_{IF}\left(t\right)=\sum_{m=1}^{L}\sqrt{2A_{J,m}}\cos\left[2\pi \left(f_{ins,m}\left(t\right)\right)t+\theta_{J,m}\right]$$

- $A_{J,m}$ is the power of the *m*th jamming signal;
- $f_{ins,m}\left(t\right)$ is the *m*th jamming instantaneous frequency;
- $\theta_{J,m}$ is the phase delay of the *m*th jamming signal;
- L is the number of interferences.

For the linear chirp signal, the instantaneous frequency $f_{ins,m}\left(t\right)$ linearly evolves with the time interval $\left[f_{begin},f_{end}\right]$. Therefore, the $f_{ins,m}\left(t\right)$ is written as follows

(4) $$f_{ins,m}\left(t\right)=f_{begin}+kt,\;0\leq t\leq T_{j}$$

where $T_{j}$ is the sweep period, $f_{begin}$ is the initial frequency, and k is the frequency change rate, also named chirp rate, defined as follows

(5) $$k=\frac{f_{end}-f_{begin}}{T_{j}}=\frac{B_{sweep}}{T_{j}}$$

where $B_{sweep}$ represents the sweep bandwidth. The *m*th jamming power to the *l*th GNSS signal power ratio (JSR) is written as follows

(6) $$JSR=10\log_{10}\left\{\frac{A_{J,m}}{A_{l}}\right\}$$

The jamming power to noise power ratio (JNR) for the *m*th jamming signal is written as follows

(7) $$JNR=10\log_{10}\left\{\frac{A_{J,m}}{N_{0}B_{IF}}\right\}$$

where $B_{IF}$ is the RF front-end bandwidth. The analytical expression of the received signal in Equation (1) is defined as follows

(8) $$X_{a}\left(t\right)=X_{IF}\left(t\right)+j{\hat{X}}_{IF}\left(t\right)$$

where the ${\hat{X}}_{IF}\left(t\right)$ is the Hilbert transform of $X_{IF}\left(t\right)$; the use of the analytic signal $X_{a}\left(t\right)$ can avoid the presence of cross-terms which could be generated by the interaction between positive and negative frequency components [19].

## 3. The Proposed Method

**Figure 1:**
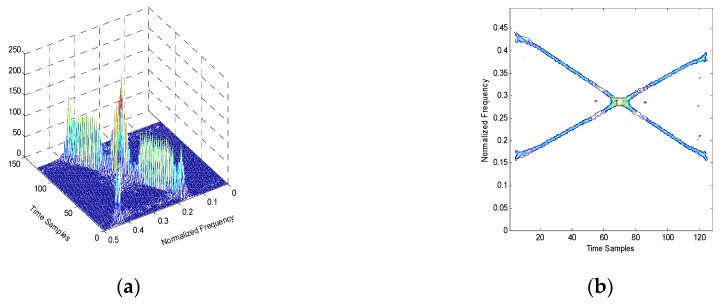
The RSPWVD of two chirps with crossed frequency. (**a**) Mesh of RSPWVD. (**b**) Contour of RSPWVD.

When it comes to the time-varying interference, the classical time-frequency analysis based on WVD and RSPWVD provides superior performance. However, WVD suffers from cross-term interference seriously when the analytic signals have two or more components [20]. The RSPWVD can reduce the cross-term interference, but it cannot deal with the signals with crossed or overlapped frequency by the peak detection method [18]. An example of RSPWVD with two chirps whose frequencies are crossed is shown as Figure 1. Figure 1a depicts that the peaks of two chirps are clear except the overlapped frequency part. In Figure 1b, it is obvious that the outline of two chirps in the overlapped frequency is blurred so that it is difficult to distinguish which signal the frequency of the overlapped part belongs to. Therefore, the proposed method based on Hough transform of RSPWVD is introduced [21,22,23].

### 3.1. Hough Transform

**Figure 2:**
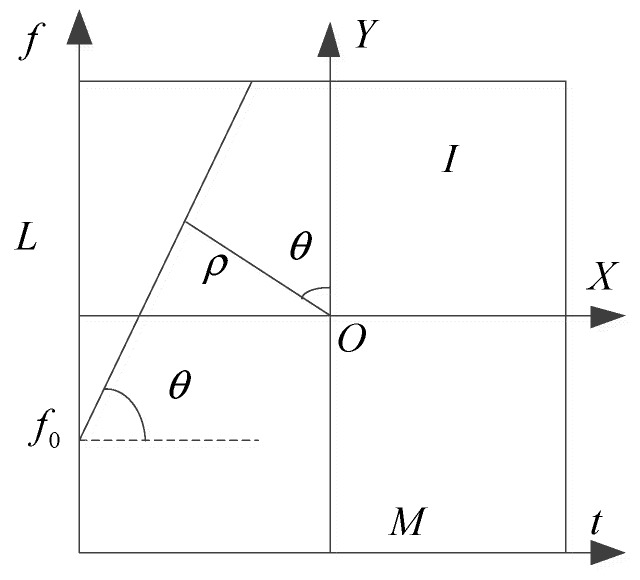
Hough transform diagram.

Hough transform has been widely used in line detection problems in images. In image I, as shown in Figure 2, the X-Y coordinate origin is at the center of the image with size M×L, then (x,y) is satisfied as follows

(9) $$\begin{array}{l} x=t-\frac{M}{2}\\ y=f-\frac{L}{2} \end{array}$$

where f and t represent the respective frequency variable and time variable in the TF plane. Equation (9) in polar coordinates can be rewritten

(10) xcosθ+ysinθ=ρ

### 3.2. The Combination of WVD and Hough Transform

WVD produces an energy distribution concentrated along a straight line. As a result, the problem of detecting the chirp interference can be turned into an issue of detecting the TF plane line and performed by Hough transform, which has been widely used for detecting chirp signal combined with the TF distribution [22,23]. The combination of WVD and Hough transform can be defined as follows

(11) $$WH_{x}\left(f_{0},k\right)=\int_{-\infty }^{\infty }\int_{-\infty }^{\infty }X_{a}\left(t+\frac{\tau }{2}\right)X_{a}^{*}\left(t-\frac{\tau }{2}\right)e^{-j2\pi \left(f_{0}+kt\right)\tau }d\tau dt$$

where $X_{a}\left(t\right)$ is an analytical signal and $X_{a}^{*}\left(t\right)$ is a complex conjugate of $X_{a}\left(t\right)$, $f_{0}$ is the initial frequency, k is the chirp rate. Both k and $f_{0}$ can be deduced from Figure 2 by a geometric relationship, the results can be written

(12) $$f_{0}=\left[\frac{L}{2}-\frac{M}{2}\tan\theta +\frac{\rho }{\cos\theta }\right]\Delta f$$

(13) $$k=\tan\theta \frac{\Delta f}{\Delta t}$$

where Δf is the frequency resolution, which equals $\frac{F_{s}}{2M}$ while Δt is the time resolution and equal to $\frac{1}{F_{s}}$, $F_{s}$ is the sampling rate. The Hough transform of WVD maps the point (t,f) of the TF plane line onto the point (ρ,θ) in the parameter plane. As a consequence, a peak is formed at the point (ρ,θ) of the parameter plane. Once the peak value exceeds a predetermined threshold, it can be determined that there is a chirp interference.

### 3.3. Double Threshold Detection

This paper adopts the double threshold detection method named primary threshold and secondary threshold [24]. The GNSS signals are buried in thermal noise assumed to be zero mean, independent and identically distributed (IID). When interference is absent, the complex random variable $X_{a}\left(t\right)$ in Equation (8) is the zero mean and IID. Its magnitude spectrum ϕ(k,l) in the TF plane can be written [25]

(14) ϕ(k,l)=Re2[Xa(tk,fl)]+Im2[Xa(tk,fl)]

where k=1,⋯,N; l=1,⋯,N; N is the length of samples. $X_{a}\left(t_{k},f_{l}\right)$ is the result of TF transform for $X_{a}\left(t\right)$. The variable |ϕ(k,l)| follows a Rayleigh distribution. The primary threshold setting is written as [26]

(15) $$\eta =\bar{\phi \left(k\right)}\sqrt{\frac{4}{\pi }}\sqrt{-\ln\left(P_{false}\right)}$$

where

(16) $$\bar{\phi \left(k\right)}=\frac{1}{N}\sum_{l=1}^{N}\left|\phi \left(k,l\right)\right|$$

$\bar{\phi \left(k\right)}$ is the average mean of ϕ(k,l), $P_{false}$ is the false alarm rate. From Equation (15), the primary threshold can be determined by a predefined $P_{false}$. Then, the data in $X_{a}\left(t_{k},f_{l}\right)$ are separated to a new set which is higher than the primary threshold and the Hough transform is applied to these data. The secondary threshold in the Hough domain is used to detect the target peak and is based on the primary threshold. The $P_{false}$ is determined by both the primary threshold and secondary threshold, written as

(17) $$P_{false}=f\left(\eta ,\varepsilon \right)$$

where η is the primary threshold and ε is the secondary threshold. When the primary threshold is determined by the initial probability of false alarm, the secondary threshold is set by Monte Carlo simulation with fixed $P_{false}$.

### 3.4. Joint Method Based on Hough Transform of RSPWVD

**Figure 3:**
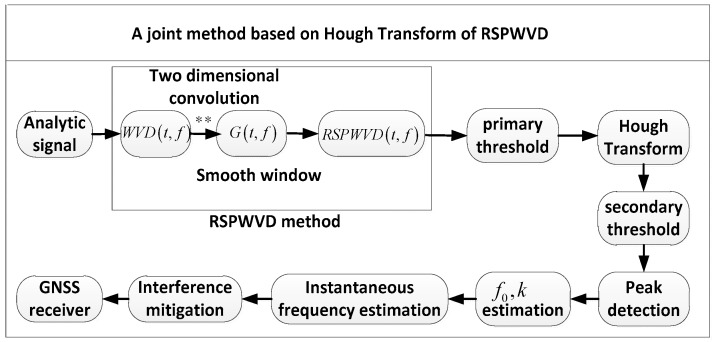
Block diagram of the joint method based on the Hough transform of RSPWVD to detect chirp interferences.

From the above analysis, not only WVD but also RSPWVD can be combined with Hough transform. However, WVD suffers from severe cross-term interference. As a result, it will affect the result of the Hough transform. Therefore, the Hough transform of RSPWVD is proposed [23]. The general expression of the Hough transform of the time-frequency transform can be written as

(18) $$TFH_{X}\left(f_{0},k\right)=\int_{-\infty }^{\infty }TF_{X}\left(t,f_{0}+kt\right)dt$$

where $TFH_{X}\left(f_{0},k\right)$ is the TF distribution of the analytical signal $X_{a}\left(t\right)$. From Equation (10), the chirp interference in the TF plane is written as polar coordinates as well as Cartesian coordinates. Therefore, Equation (11) can be written as

(19) $$TFH_{X}\left(\rho ,\theta \right)=\int_{-\infty }^{\infty }TF_{X}\left(t,f\left(\rho ,\theta \right)\right)dt$$

where f(ρ,θ) is the instantaneous frequency of the chirp interference in polar coordinates. Equation (18) as well as Equation (19) depicts that the line of the TF plane becomes a peak at point (ρ,θ) of the parameter plane by Hough transform. Conversely, a peak in the parameter plane represents a line in the TF plane and can be used to estimate the parameter of the line. As a result, this can be used to detect and identify chirp interferences. The joint method based on the Hough transform of RSPWVD is displayed as Figure 3 and is based on the following steps.

1. map $X_{a}\left(t\right)$ onto the TF plane by computing its RSPWVD
  (20) $$X_{a}\left(t\right)↔RSPWVD_{X}\left(t,f\right)$$
2. separate the data of $RSPWVD_{X}\left(t,f\right)$ into two sets and map the set above the primary threshold onto the Hough transform.
  (21) $$RSPWVD_{X}\left(t,f\right)↔TFH_{X}\left(\rho ,\theta \right)$$
3. search the peaks of $TFH_{X}\left(\rho ,\theta \right)$; if a peak exceeds the secondary threshold, record its point value (ρ,θ)
4. estimate the parameter $\left(f_{0},k\right)$ by the point value (ρ,θ)
  (22) $$TFH_{X}\left(\rho ,\theta \right)↔TFH_{X}\left(f_{0},k\right)$$

### 3.5. Impact on the Acquisition Stage

In the case of a single satellite and a single chirp interference, the input signal $X_{IF}\left(t\right)$ in Equation (1) enters the ADC without considering the quantization effect and can be rewritten as

(23) $$X_{IF}\left(n\right)=\sqrt{2A}C\left(n-\tau \right)D\left(n-\tau \right)\cos\left[2\pi \left(f_{IF}+f_{d}\right)nT_{s}+\varphi_{IF}\right]+\sqrt{2A_{J}}\cos\left[2\pi \left(f_{ins}\left(t\right)\right)nT_{s}+\theta_{J}\right]+N\left(n\right)$$

Then, the signal $X_{IF}\left(n\right)$ is multiplied by two orthogonal sinusoids as well as a local signal replica and is integrated; the results are as follows [27]

(24) $$\begin{array}{l} S_{I}\left(\tau ,f_{D}\right)=\frac{1}{N}\sum_{n=0}^{N-1}r_{I}\left(n\right)c\left(n-\tau \right)=r_{I}\left(\tau \right)*h_{c}\left(\tau \right)\\ S_{Q}\left(\tau ,f_{D}\right)=\frac{1}{N}\sum_{n=0}^{N-1}r_{Q}\left(n\right)c\left(n-\tau \right)=r_{Q}\left(\tau \right)*h_{c}\left(\tau \right) \end{array}$$

where $r_{I}\left(n\right)=X_{IF}\left(n\right)\cos\left(2\pi f_{D}n\right)$, $r_{Q}\left(n\right)=X_{IF}\left(n\right)\sin\left(2\pi f_{D}n\right)$, $f_{D}=\left(f_{IF}+f_{d}\right)T_{s}$, (∗) denotes integral operation, *N* is the length of local code and $h_{c}\left(\tau \right)$ is an equivalent filter. The Cross Ambiguity Function (CAF) is obtained [27]:

(25) $$S\left(\tau ,f_{D}\right)=\sqrt{S_{I}{\left(\tau ,f_{D}\right)}^{2}+S_{Q}{\left(\tau ,f_{D}\right)}^{2}}$$

When the Doppler shift $f_{d}$ and the code delay τ are correctly recovered, the detection probability that the statistical variable $S\left(\tau ,f_{D}\right)$ surpasses a fixed threshold β in the GNSS acquisition stage can be defined:

(26) $$P_{\det}\left(\beta \right)=P\left(S\left(\tau ,f_{D}\right)>\beta \right)$$

In order to assess the impact of chirp interference on the detection probability, it is supposed that the GNSS signal and the chirp interference are known. The integration results of GNSS signals in Equation (24) can be written [27]:

(27) $$S_{y}\approx \sqrt{C/2}\exp\left\{-j\varphi_{IF}\right\}$$

Similarly, the integration results of chirp interference in Equation (24) can be written [27]:

(28) $$S_{J}=k_{1}\sqrt{\frac{A_{J}}{2}}\exp\left\{j2\pi \left(f_{\mathrm{in}s}+f_{D}\right)\tau +j\theta_{J}+j\theta_{1}\right\}+k_{2}\sqrt{\frac{A_{J}}{2}}\exp\left\{-j2\pi \left(f_{\mathrm{in}s}-f_{D}\right)\tau -j\theta_{J}+j\theta_{2}\right\}$$

where $\theta_{1}=∠\left\{H_{c}\left(f_{ins}+f_{D}\right)\right\}$ and $\theta_{2}=∠\left\{H_{c}\left(-f_{ins}+f_{D}\right)\right\}$. $H_{c}\left(f\right)$ is the Fourier transform of $h_{c}\left(n\right)$. $k_{1}$ and $k_{2}$ can be written as

(29) $$k_{i}^{2}={\left|H_{c}\left(\pm f_{ins}+f_{D}\right)\right|}^{2}={\int_{-\infty }^{\infty }\left|H_{c}\left(f\right)\right|}^{2}\delta \left(f-\left(\pm f_{ins}+f_{D}\right)\right)df=\int_{-\infty }^{\infty }G_{s}\left(f\right)G_{i}\left(f\right)df$$

where δ(·) is the delta Dirac, $G_{s}\left(f\right)={\left|H_{c}\left(f+f_{D}\right)\right|}^{2}$ and $G_{i}\left(f\right)=\delta \left(f\pm f_{ins}\right)$. When the chirp interference appears, the CAF in Equation (25) follows a Rice distribution [27]

(30) $$S\left(\tau ,f_{D}\right)=\frac{x}{\sigma^{2}}\exp\left\{-\frac{x^{2}+\alpha^{2}}{2\sigma^{2}}\right\}I_{0}\left(\frac{x\alpha }{\sigma^{2}}\right),\;x>0$$

where $\alpha^{2}={\left|S_{y}+S_{J}\right|}^{2}$, $\sigma^{2}=\frac{\sigma_{out}^{2}}{2}$, $\sigma_{out}^{2}$ is the noise variance of ouput of equivalent filter $h_{c}\left(n\right)$ and $\sigma_{out}^{2}=\frac{1}{N}N_{0}B_{IF}$, $I_{0}$ is the modified Bessel function with first kind and zero order. The detection probability can be defined:

(31) $$P_{d}\left(\beta \right)=\int_{\beta }^{\infty }\frac{x}{\sigma^{2}}\exp\left\{-\frac{x^{2}+\alpha^{2}}{2\sigma^{2}}\right\}I_{0}\left(\frac{x\alpha }{\sigma^{2}}\right)dx=Q\left(\frac{\alpha }{\sigma };\frac{\beta }{\sigma }\right)$$

where Q(⋅;⋅) denotes the Marcum Q function. From Equation (31), the chirp interference seriously affects the detection probability in the GNSS acquisition stage.

## 4. Results

To test the performance of the introduced method, an experiment under the condition where the BDS B1I signals are corrupted by two simulation chirps is carried out for several scenarios. The main hardware parameters of the down converter and ADC are recorded in Table 1.

**Figure 4:**
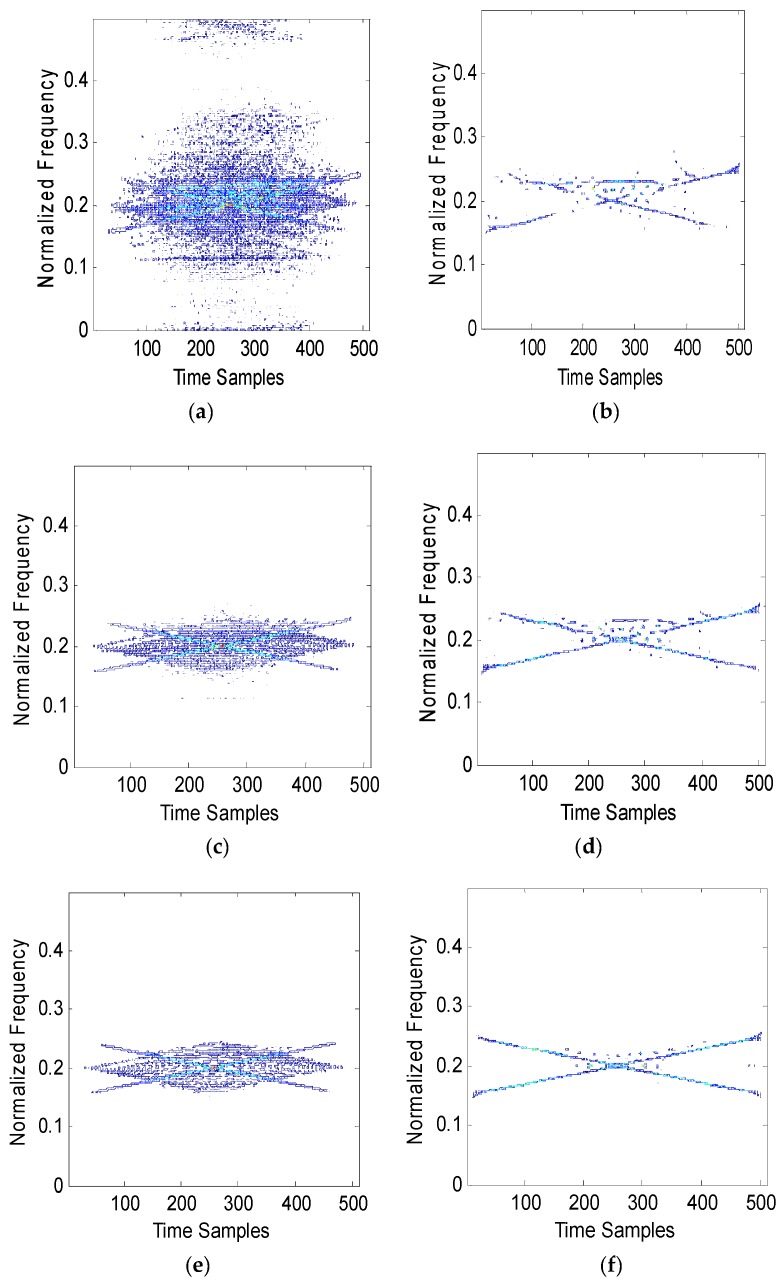
The WVD and RSPWVD of BDS B1I signals with two chirps, $C_{0}/N$ is 43 dB-Hz and the JNR is 0 dB, 6 dB and 12 dB. (**a**) WVD, JNR is 0 dB. (**b**) RSPWVD, JNR is 0 dB. (**c**) WVD, JNR is 6 dB. (**d**) RSPWVD, JNR is 6 dB. (**e**)WVD, JNR is 12 dB. (**f**) RSPWVD, JNR is 12 dB.

First, the JNR is set to 0 dB, 6 dB and 12 dB. The instantaneous frequency of one chirp interference is from 50 MHz to 30 MHz, and the instantaneous frequency of the other chirp interference is from 30 MHz to 50 MHz. The sweep period is 2.56 us and the sample length is 512. The Hough transforms combined by WVD and RSPWVD are shown as Figure 4.

Figure 4a shows that the WVD of two chirps suffers from cross-term interferences seriously due to the interaction of different chirp signal components; the outline of two chirps are not clear. Figure 4b depicts the RSPWVD of two chirps, the outline of which are clear. The cross-terms are less than those in Figure 4a. Figure 4c presents the WVD of chirps with a higher JNR and its energy distribution is clear and suffers from less cross-term interferences compared with Figure 4a. Figure 4d represents a clear RSPWVD which suffers from fewer cross-term interferences compared with that in Figure 4c. Although the power of chirps is 12 dB above that of noise, the WVD of chirps in Figure 4e still suffers from cross-term interference, while in Figure 4f, the outline of RSPWVD is very clear and its energy distribution hardly suffers from cross-term interference.

**Figure 5:**
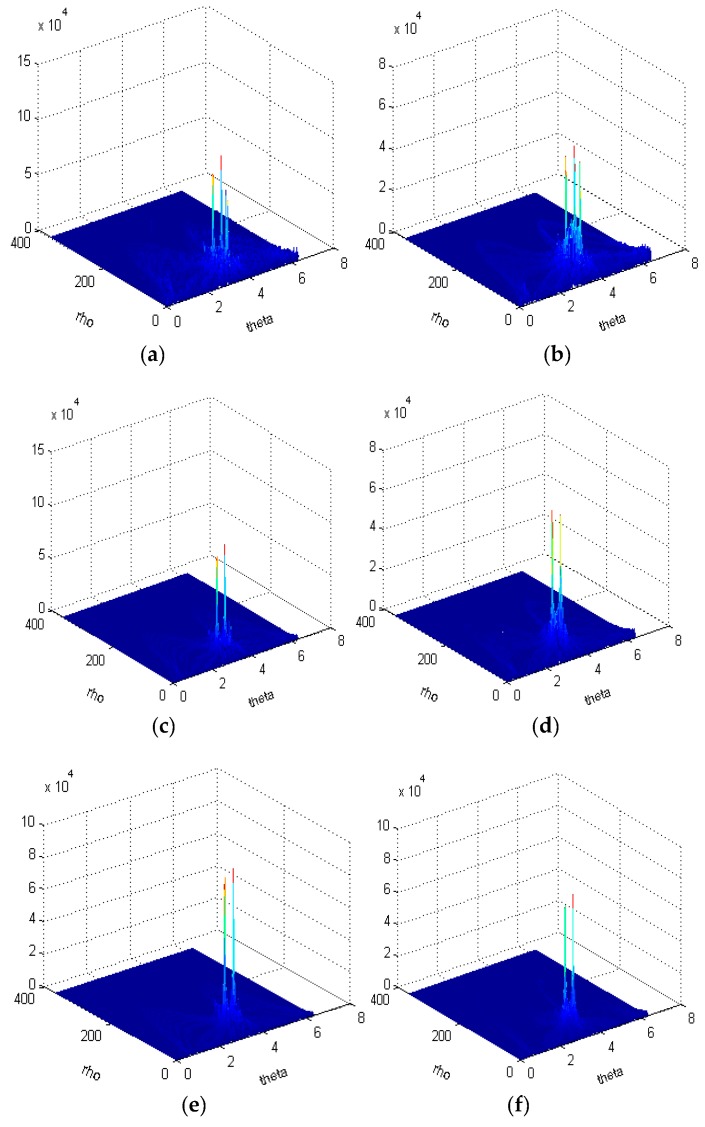
The Hough transform of WVD and RSPWVD with BDS B1I signals corrupted by two chirps, $C_{0}/N$ is 43 dB-Hz. (**a**) Hough transform of WVD, JNR is 0 dB. (**b**) Hough transform of RSPWVD, JNR is 0 dB. (**c**) Hough transform of WVD, JNR is 6 dB. (**d**) Hough transform of RSPWVD, JNR is 6 dB. (**e**) Hough transform of WVD, JNR is 12 dB. (**f**) Hough transform of RSPWVD, JNR is 12 dB.

However, Figure 4 shows that the common frequency (overlapped frequency) which belongs to chirp one as well as chirp two cannot be identified by the peak detection method. As a result, it is difficult to estimate instantaneous frequency in overlapped parts for each chirp. In order to deal with this problem, the experiment with the condition where the Hough transform is combined with WVD and RSPWVD is conducted; the experimental setup is as in Figure 4 and the results are shown in Figure 5.

Figure 5a shows the Hough transform of WVD and there are three peaks in the Hough domain. The peak on the right is the pseudo peak. It is a cross-term, which is integrated by Hough transform in the TF plane and formed into a peak. What is shown in Figure 5b is similar to that in Figure 5a. Although the RSPWVD can reduce cross-term interferences, the power of chirps is as strong as that of noise. As a result, the RSPWVD reallocates the noise energy which is integrated by the Hough transform in the TF plane and is formed into a strong peak. Figure 5c depicts two strong peaks and a weak pseudo peak, while in Figure 5d there are only two strong peaks without a pseudo peak. In Figure 5e,f, there are two peaks without a pseudo peak. Figure 5 depicts that although the WVD suffers from cross-term interferences, the Hough transform can help to reduce the effect of the cross-term interferences when the chirp signals are strong. In addition, the RSPWVD can eliminate the cross-term interference at the expense of its good localization and concentration properties; the combination of RSPWVD and Hough transform can effectively reduce the effect of cross-term interferences.

**Figure 6:**
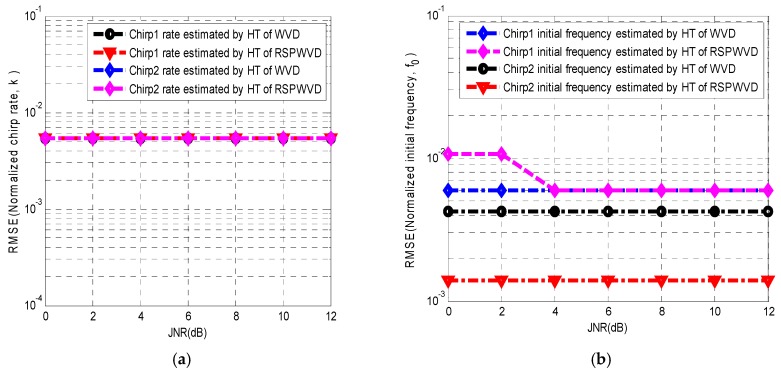
RMSE of the chirp rate and initial frequency estimation for chirp interferences present in BDS B1I signals by Hough transform combined by WVD and RSPWVD versus JNR. The $C_{0}/N$ is 43 dB-Hz. (**a**) RMSE of chirp rate. (**b**) RMSE of initial frequency.

In order to assess the performance between the Hough transform of WVD and RSPWVD, the root mean square errors (RMSE) of the rate estimation and initial frequency estimation for chirp interference are used and displayed as a function of JNR in Figure 6. The setting parameters of the experiments are the same as in Figure 4. Figure 6a shows that the RMSE of the chirp one rate estimated by the Hough transform of WVD is the same as that estimated by the Hough transform of RSPWVD when JNR ranges from 0 dB to 12 dB. Similarly, the RMSE of the chirp two rate estimated by the Hough transform of WVD is the same as that estimated by the Hough transform of RSPWVD when JNR ranges from 0 dB to 12 dB. In Figure 6a, it is obvious that the Hough transform of RSPWVD as well as that of WVD provides the same RMSE of the rate estimation for each chirp when JNR ranges from 0 dB to 12 dB.

In Figure 6b, the RMSE of the initial frequency for chirp one estimated by the Hough transform of RSPWVD is close to $10^{-2}$ when JNR is equal to 0 dB and 2 dB, while the RMSE of the initial frequency for chirp one estimated by the Hough transform of WVD is about $0.5\times 10^{-2}$ when JNR is equal to 0 dB and 2 dB. However, when JNR equals or surpasses 4 dB, the Hough transform of RSPWVD and WVD provides the same RMSE of the initial frequency for chirp one. On the other hand, the RMSE of the initial frequency for chirp two estimated by the Hough transform of WVD is close to $0.4\times 10^{-2}$ when JNR ranges from 0 dB to 12 dB. The RMSE of the initial frequency for chirp two estimated by the Hough transform of RSPWVD is close to $0.1\times 10^{-2}$ when JNR ranges from 0 dB to 12 dB. Figure 6b depicts that the Hough transform of RSPWVD offers a poorer RMSE of the initial frequency estimation for chirp one compared with the Hough transform of WVD when JNR is below 4 dB and provides the same RMSE of the initial frequency estimation as that of WVD for chirp one when JNR equals or surpasses 4 dB. For chirp two, the proposed method provides a better RMSE of the initial frequency estimation compared with the Hough transform of WVD.

In addition, another experiment is performed by the proposed method under the condition where one chirp is adopted and its instantaneous frequency ranges from 30 MHz to 50 MHz. The sample length is 512 and the sweep period is 2.56 us, 5.12 us and 10.24 us. The results of RMSE are shown in Table 2. In Table 2, the estimation of initial frequency is close to $0.5\times 10^{-2}$ and the estimation of the chirp rate is about $0.6\times 10^{-2}$ when the sweep period is 2.56 us. However, when the sweep period increases to 5.12 us, the estimation of the initial frequency decreases by approximately $1.5\times 10^{-2}$, and the estimation of chirp rate decreases by almost $0.7\times 10^{-2}$. Finally, when the sweep period reaches 10.24 us, the estimation of initial frequency continues to decline by about $1.7\times 10^{-2}$ and the estimation of the chirp rate reduces to a level near $0.8\times 10^{-2}$. From Table 2, it is obvious that the estimation accuracy of the initial frequency as well as the chirp rate declines as the sweep period increases.

The computational requirements of the Hough transform of WVD and RSPWVD are shown in Table 3 [18]. N is sample length, M is the length of the time window and L is the length of the frequency window. The computational requirements of the Hough transform of WVD consist of the computational requirements of the Hough transform as well as those of WVD. The output time-frequency matrix of WVD is N×N, the discrete points of θ from 0 to 2π are K. From Equation (10), the Hough transform needs two complex multiplications and one complex addition. Therefore, the total requirements of the Hough transform are $2N^{2}K$ complex multiplications and $N^{2}K$ complex additions. Similarly, the total requirements of the Hough transform in case of RSPWVD are 2NMK complex multiplications and NMK complex additions. From Table 3, the requirements of WVD are higher than those of RSPWVD because of N≫M and N≫L. As a result, the tatal requirements of the Hough transform of WVD are higher than those of RSPWVD.

**Figure 7:**
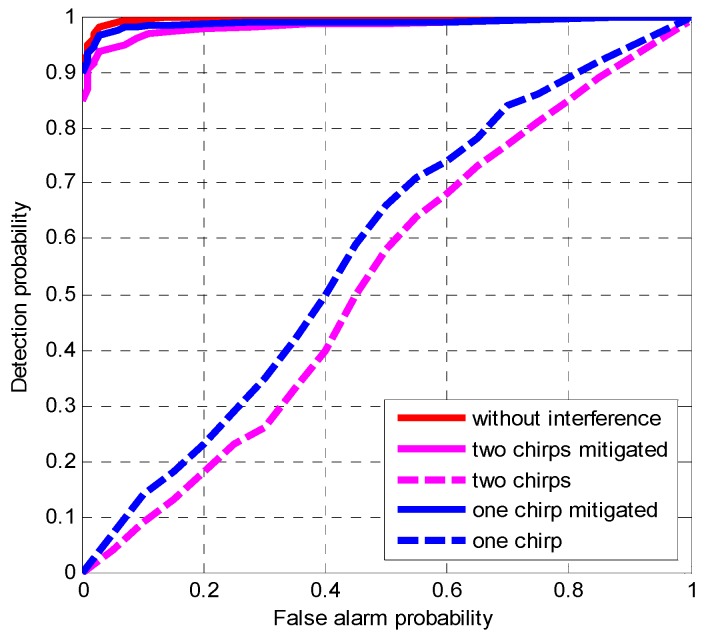
ROC curves for BDS B1I signals affected by chirp interference. The JNR is 12 dB, the $C/N_{0}$ is 43 dB-Hz. The coherent integration time is 1 ms.

Finally, the chirp signal is a continuous wave at any fixed time and can be mitigated by a notch filter based on the second-order direct form structure with two parameters named α and β which are determined by the power of chirp interference and the instantaneous frequency estimated by proposed method, respectively. The receiver operating characteristic (ROC) curves are shown in Figure 7 by Monte Carlo simulations to analyze the performance of the notch filter to mitigate the chirp interference under the condition where the BDS B1I signals are simulated with chirp interferences. The JNR is set to 12 dB and the $C/N_{0}$ is set to 43 dB-Hz. The integration time is set to 1 ms. The instantaneous frequency of one chirp ranges from 30 MHz to 50 MHz and the instantaneous frequency of the other ranges from 50 MHz to 30M Hz. The sweep period is 2.56 us.

Figure 7 depicts that the ROC curve in the two chirp scenario presents a worse acquisition performance than that in the one chirp scenario. However, when the notch filter is adopted, the ROC curves present an improved acquisition performance. The results depicts that the proposed method can effectively detect the one or two chirp interferences, especially for those with crossed frequency without a priori knowledge.

## 5. Conclusions

This paper presents a joint method based on the Hough transform of RSPWVD to detect time-varying interferences with crossed frequency for GNSS receivers with a single antenna. The analytical expression of initial frequency estimation and chirp rate estimation is presented and the double threshold detection is proposed as well.

The performance of the developed method has been assessed by experiments under conditions where the real BDS B1I signals corrupted by the simulated chirp interferences are collected by the GNSS software receiver. The actual performance of experiments has been shown by the quantitative metric RMSE of the parameter estimation. In addition, the effect of the sweep period on the estimation of the initial frequency and chirp rate has been analyzed.

The ROC curves have been used to assess the performance of the notch filter to mitigate chirp interference by Monte Carlo simulations. The results depict that the proposed method can effectively detect and identify the chirp interferences with crossed frequency and provide the same root mean square errors (RMSE) of the parameter estimation for chirp one and the improved initial frequency estimation for chirp two compared with the Hough transform of WVD when JNR equals or surpasses 4 dB. Furthermore, the RSPWVD method itself can provide better performance in reducing cross-term interference and needs less computational requirements compared with the WVD method.

## Figures and Tables

**Table 1:** Down-converter digitizer parameters.

Parameter	Value
Bandwidth	20 MHz
Intermediate frequency	40 MHz
Down Converter Gain	60 dB
Dynamic range in Down Converter	70 dB
Sampling Rates	200 MHz
Bits Per sample	14

**Table 2:** Chirp detection for different sweep periods.

Sweep Period (us)	Estimation of Initial Frequency RMSE (Normalized)	Estimation of Chirp Rate RMSE (Normalized)
2.56	0.005438	0.005941
5.12	0.015086	0.006581
10.24	0.017463	0.007522

**Table 3:** Computational requirements of the Hough transform of WVD and RSPWVD.

Method	Computational Requirements
WVD	$2N^{2}+2N^{2}\log^{2N}$ complex multiplications $4N^{2}\log^{2N}$ complex additions
RSPWVD	$NM\left(2+L\right)+NM/2\log^{M}$ complex multiplications $NM\left(4+L\right)+NM\log^{M}$ complex additions
WVD + Hough	$2N^{2}+2N^{2}\log^{2N}+2N^{2}K$ complex multiplications $4N^{2}\log^{2N}+N^{2}K$ complex additions
RSPWVD + Hough	$NM\left(2+L\right)+NM/2\log^{M}+2NMK$ complex multiplications $NM\left(4+L\right)+NM\log^{M}+NMK$ complex additions
